# A new biological triangle in cancer: intestinal microbiota, immune checkpoint inhibitors and antibiotics

**DOI:** 10.1007/s12094-021-02659-w

**Published:** 2021-06-14

**Authors:** Jie Zhang, Zhujiang Dai, Cheng Yan, Wenjie Zhang, Daorong Wang, Dong Tang

**Affiliations:** 1 Clinical Medical College, Yangzhou University, Yangzhou, Jiangsu Province China; 2 Dalian Medical University, Dalian, China; 3 Department of General Surgery, Institute of General Surgery, Clinical Medical College, Northern Jiangsu Province Hospital, Yangzhou University, Yangzhou, 225001 China

**Keywords:** Immunotherapy, Immune checkpoint inhibitors, Antibiotics, Microbiota, Cancer

## Abstract

Cancer immunotherapy has revolutionized the treatment of many malignant tumors. Although immune checkpoint inhibitors (ICIs) can reactivate the anti-tumor activity of immune cells, sensitivity to immune checkpoint inhibitor therapy depends on the complex tumor immune processes. In recent years, numerous researches have demonstrated the role of intestinal microbiota in immunity and metabolism of the tumor microenvironment, as well as the efficacy of immunotherapy. Epidemiological studies have further demonstrated the efficacy of antibiotic therapy on the probability of patients' response to ICIs and predictability of the short-term survival of cancer patients. Disturbance to the intestinal microbiota significantly affects ICIs-mediated immune reconstitution and is considered a possible mechanism underlying the development of adverse effects during antibiotic-based ICIs treatment. Intestinal microbiota, antibiotics, and ICIs have gradually become important considerations for the titer of immunotherapy. In the case of immunotherapy, the rational use of antibiotics and intestinal microbiota is expected to yield a better prognosis for patients with malignant tumors.

## Introduction

Cancer immunotherapy has been significant in the management of various malignant tumors. One of the key therapies involves the use of immune checkpoint inhibitors (ICIs), which enhance the anti-tumor activity of immune cells by blocking specific immune checkpoints, such as programmed death receptor 1/programmed death-ligand 1(PD-1/PD-L1), and cytotoxic T lymphocyte antigen 4 (CTLA-4), among others. Recent reports show that the tumor microenvironment is a simple sterile but complex environment rich in the microbiota [1]. A study revealed that differences in human lifestyles and physiological variables exert varying effects on intestinal microbiota, hence provide potentially important clues to human health [2]. Also, numerous studies have found an association of microbiota with human immunity and metabolism, particularly in the regulation of response to cancer immunotherapy [3, 4]. For example, *Bifidobacteria* was found to induce activation of dendritic cells and promote proliferation of tumor-specific CD8^+^ T cells, thereby exerting anti-tumor immune effects [5]. These pieces of evidence affirm that intestinal microbiota plays a critical role in the immunotherapy of cancer patients. However, immune-related adverse events caused by immunotherapy have been associated with the amplification of existing symptoms of infection due to malignant tumors or treatment-related immunosuppression [6]. Overall, the status of antibiotics in the treatment of tumors is beyond doubt. Although intestinal microbiota can optimize the efficacy of ICIs in tumor management [7], information regarding the impact of broad-spectrum antibiotics on the intestinal commensal microbiota is scanty. Herein, we reviewed the correlation between intestinal microbiota, antibiotics, and immunotherapy. For effective immunotherapy, clinicians should recommend the rational use of antibiotics and intestinal microbiota to improve the efficacy of ICIs. These insights will guide immunotherapeutic strategies and improve patient prognosis.

## Immune checkpoint mechanisms of PD-1/PD-L1 and CTLA-4 in cancer immunotherapy

### PD-1/PD-L1

**Fig. 1:**
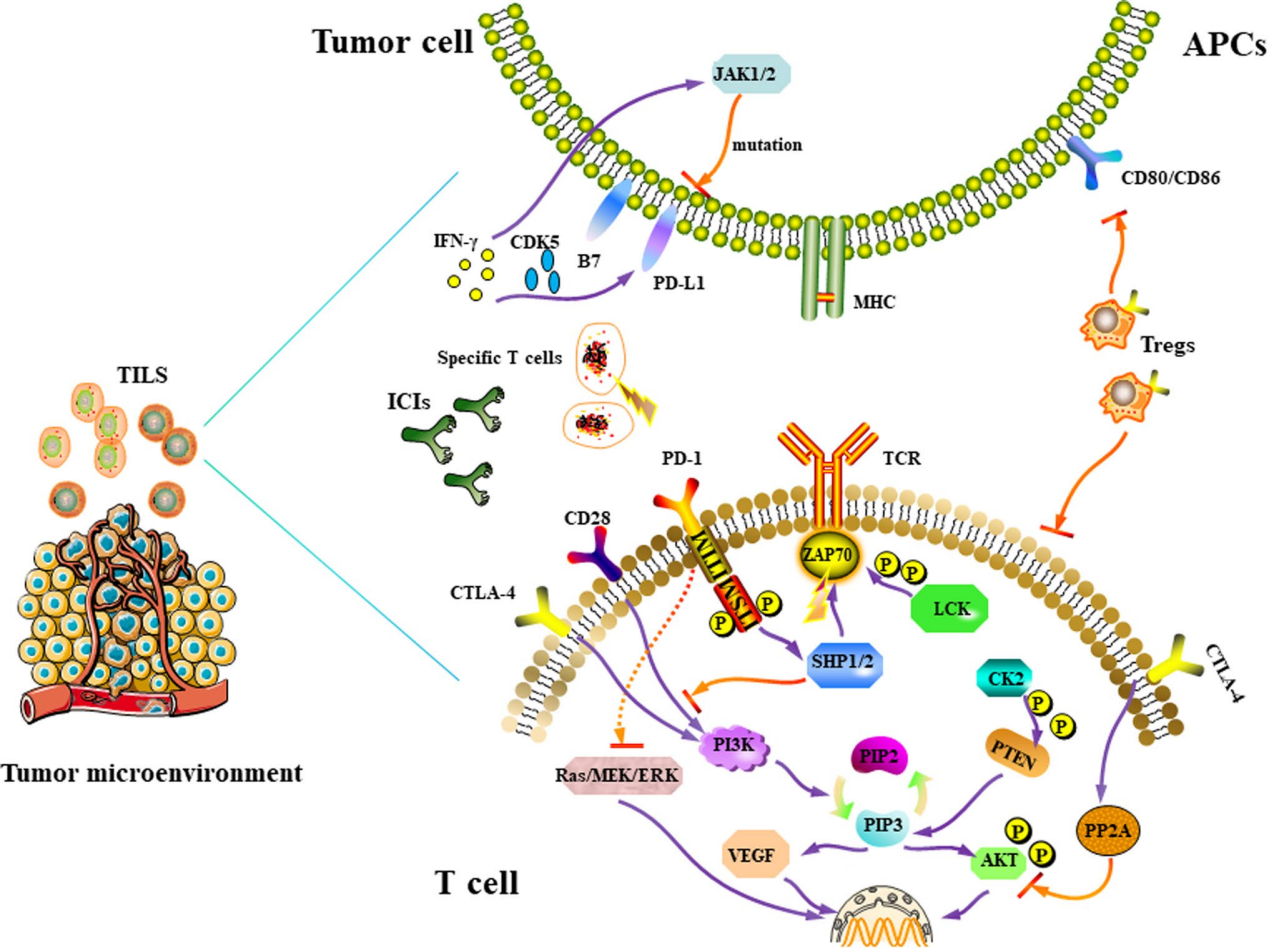
The signaling pathway of immune checkpoint PD-1/PD-L1 and CTLA-4 in cancer immunotherapy. When PD-L1 of tumor cells recognizes PD-1 on the surface of immune cells, ITSM phosphorylates and activates SHP-1/SHP-2. This process inactivates the TCR adaptor molecule ZAP70 and blocks the CD28-mediated PI3K pathway, thereby inhibiting T cell activation. ZAP70 is also phosphorylated by LCK, and further inhibits the expression of the signaling pathway. Another mechanism through which PD-1 inhibits TCR signaling is through blockade of CK2-mediated phosphorylation of phosphatase and PTEN. PIP3 can cause phosphorylation of AKT and send a signal to the nucleus. Activation of the PI3K/AKT/mTOR and Ras/MEK/ERK signaling pathways is also inhibited in the process. Therefore, PD-1 can inhibit downstream immune pathways of tumors through PI3K and Ras. A combination of CTLA-4 and B7 may produce inhibitory signals, including inhibition of TCR immune synapses and increasing T cell fluidity, thereby avoiding mutual recognition with APCs. Moreover, CTLA-4 mediated PP2A can inhibit AKT and suppress IL-2 production, while Tregs expressing CTLA-4 have extracellular inhibitory effects on common T cells. Therefore, CTLA-4 mainly helps tumors to evade immune surveillance by competitively binding to B7 ligands and inhibiting the function of Tregs

In the tumor microenvironment, activation of the PD-1/PD-L1 pathway plays a crucial role in the immune escape process of tumors. PD-1, a protein in the CD28 family, contains an immunoreceptor tyrosine-based switch motif (ITSM) and an immunoreceptor tyrosine-based inhibitory motif (ITIM) which primarily exists on the surface of activated immune cells, such as T/B and dendritic cells, among others [8]. On the other hand, PD-L1 is a protein in the B7 family that is expressed in antigen-presenting cells (APCs) and tumor-infiltrating cells. At the molecular level, T lymphocytes undergo full activation by two independent and simultaneous signals. First, the naive T cells must successfully recognize tumor antigens presented by APCs, and present tumor peptides associated with major histocompatibility complex (MHC) I on their surface [9, 10]. Second, a signal transduction molecule, B7, on APCs binds to CD28 and induces a second signal that is crucial in T cell activation [11]. When PD-L1 in tumor cells recognizes PD-1 on the surface of immune cells, ITSM phosphorylates and activates tyrosine phosphatase SHP-1/SHP-2 [12, 13]. This process inactivates the TCR adapter molecule, ZAP70, thereby blocking the CD28-mediated PI3K pathway and inhibiting T cell activation [14, 15]. Besides, ZAP70 is phosphorylated by LCK, which further blocks the signaling pathway [16, 17] (Fig. 1). Another mechanism through which PD-1 inhibits TCR signaling entails inhibition of CK2-mediated phosphorylation of phosphatase and tensin homolog (PTEN), which protects its phosphatase activity [18, 19]. Particularly, PTEN acts as a serine-threonine phosphatase in contrast to PI3K, converting PIP3 to inactive PIP2 via its phosphatase activity, consequently inhibiting the conduction of TCR [20]. PTEN deficiency activates PI3K and promotes the secretion of immunosuppressive CCL2 and VEGF [21, 22]. Moreover, PIP3 potentially induces AKT phosphorylation and relays signals to the nucleus [23], which subsequently inhibits activation of the PI3K/AKT/mTOR and Ras/MEK/ERK signaling pathways [24, 25]. Previous studies have shown that PI3K/AKT/mTOR and Ras/MEK/ERK pathways play an important role in anti-tumor immunity [26]. Functionally, PD-1 inhibits the immune pathway for tumors by blocking the downstream pathways through PI3K and Ras [27]. Notably, several types of tumor cells have been found to express PD-L1, especially in non-small cell lung cancer (NSCLC), melanoma, renal cell carcinoma, and leukemia tumors [28, 29].

### CTLA-4

Like PD-1, CTLA-4 is a co-suppressor molecule that belongs to the CD28 family. However, unlike PD-1, the expression of CTLA-4 is initiated in regulatory T cells following their early activation [30, 31]. This feature also determines the sequential clinical use of ICIs. The underlying mechanism through which CTLA-4 inhibits T cell activation primarily depends on the competitive acquisition of B7 ligand with CD28 [32]. When CD28 has sufficiently bound to B7, T cells proliferate and produce IL-2, thereby increasing energy metabolism and cell survival [33]. CTLA-4 has a higher affinity for B7, compared to CD28, while CTLA-4/B7’s crystal structure also affirms the close relationship between them [32]. Therefore, the relative amount of binding of CTLA-4 to B7 determines whether or not T cells will be activated. Some previous evidence had suggested that the binding of CTLA-4 to B7 potentially produces inhibitory signals, including inhibition of TCR immune synapses and increased T cell mobility, thereby evading interaction with APCs [34] (Fig. 1). Apart from competing for B7 ligands, CTLA-4 also inhibits T cell activation through an intracellular signaling cascade. Previous studies show that CTLA-4-mediated recruitment of serine/threonine phosphatase PP2A inhibits AKT and further inhibits IL-2 production [35]. Simultaneously, CTLA-4 may directly induce PI3K activation, and promote the production of anti-apoptotic factor Bcl-xL, thereby enhancing the survival of T cells [36].

Numerous studies have demonstrated that Tregs expressing CTLA-4 exert extracellular inhibitory effects on traditional T cells. Conversely, Tregs lacking CTLA-4 were found to cause a fatal autoimmune disease with similar characteristics to mice lacking CTLA-4, albeit with some degree of delayed kinetics [37]. Notably, this phenotype is not related to specific deletion of Tregs, but rather a result of upregulation of CD80 and CD86 on APCs [38]. Thus, Tregs-expressed CTLA-4 limits the availability of CD80 and CD86 on APCs, a phenomenon that inhibits traditional T cell activation in an extracellular manner. Another mechanism through which Tregs control effector T cells entails downregulation of the B7 ligand on APCs, which reduces CD28 co-stimulation. Therefore, CTLA-4 helps tumors escape immune surveillance, primarily via competitive binding to B7 ligands and inhibition of the function of Tregs.

## Immune checkpoint inhibitors (ICIs) have considerable therapeutic effects

Numerous researches on ICIs have made significant progress against the checkpoints. The current ICIs contain anti-PD-1(nivolumab, and pembrolizumab, etc.), anti-PD-L1 (durvalumab, atezolizumab, etc.), and anti-CTLA-4 (ipilimumab). Topalian et al. found that nivolumab exerted excellent anti-tumor effects against solid tumors, including advanced melanoma, and this effect was accompanied by high drug safety [39]. Results from another randomized, double-blind, phase III trial revealed that nivolumab significantly improved the overall survival rate of patients with advanced melanoma without BRAF mutations, relative to dacarbazine [40]. The use of ICIs, such as nivolumab, has also been associated with some adverse events, including fatigue, itching, and nausea. Among them, 11.7 and 17.6% of nivolumab and dacarbazine users, respectively experienced grade 3 or 4 adverse events [40]. In addition, first-line pembrolizumab monotherapy has been shown to significantly improve overall survival (OS) and progression-free survival (PFS) of patients with untreated metastatic non-small cell lung cancer, as evidenced by PD-L1 tumor proportional scores (TPS) of 50% or higher [41]. For instance, a randomized controlled phase 3 trial that compared Pembrolizumab to chemotherapy for locally advanced or metastatic NSCLC (KEYNOTE-042) found that subjects in the pembrolizumab group had significantly longer overall survival rates relative to those in the chemotherapy group (*p* = 0.0018) [41]. These KEYNOTE-042 results, which represent the primary endpoint of overall survival, affirmed the efficacy of pembrolizumab as standard first-line therapy for the management of NSCLC patients with high PD-L1 expression. In another study, Bellmunt et al. found that use of platinum double-chemotherapy in Phase 3 CheckMate 026 of nivolumab in patients with metastatic or recurrent PD-L1-positive NSCLC did not effectively improve their OS, relative to pembrolizumab [42]. The primary analysis of CheckMate 026, involving patients with a PD-L1 expression level of 5% or greater based on 28–8 antibody, revealed median OS of 14.4 and 13.2 months using nivolumab and chemotherapy, respectively, with HR of 1.02 (95% CI 0.80–1.30) [43–45]. When combined with the above-mentioned multiple clinical trials, it is evident that immunotherapy can enhance OS and PFS of NSCLC patients and improve their prognosis.

Despite the unparalleled success of PD-1 ICIs in their class of drugs, some patients still cannot be treated using this monotherapy. The original intention for attempting combination therapies is to assist cancer patients who benefit less from monotherapy. The guiding principle is to enhance the efficacy of ICIs by improving tumor antigen expression or saving immune-effect dysfunctional cells [46]. Hodi et al. reported a 2-year randomized controlled trial (Phase 2) in which they evaluated the efficacy of first-line untreated advanced melanoma using nivolumab in combination with ipilimumab alongside ipilimumab alone. Their results showed that nivolumab combined with ipilimumab was more superior to the use of ipilimumab alone [47]. Notably, Hodi et al. updated these results and revealed that first-line nivolumab combined with ipilimumab or nivolumab alone exerted long-lasting, sustained clinical benefits in patients with advanced melanoma, regardless of BRAF mutation status, while combined treatment with nivolumab was more likely to improve survival outcomes relative to nivolumab alone [48].

Generally, cancer patients are manifest many complications, which may compromise the safety of ICIs. Previously, de Malet et al. revealed that ICIs could influence gastrointestinal function, causing diarrhea and colitis [49]. Notably, the resultant symptoms were either acute or subacute. Similarly, a meta-analysis of ICIs found that combined use of ipilimumab with nivolumab may cause risk of immune-related endocrine diseases [50], while Gu et al. also acknowledged that the incidence of adverse reactions is high following the use of combination therapy, and may impair treatment [51]. However, when compared to treatment-related mortality, a suitable combination regimen is still recommended. Previous evidence shows that immunotherapy has numerous advantages over traditional surgical treatment and chemoradiotherapy with regards to prolonging overall survival and improving recurrence-free survival [52, 53], affirming the prospect of using ICIs in cancer treatment [54, 55].

## Intestinal microbiota participates in immune regulation in immunotherapy

**Fig. 2:**
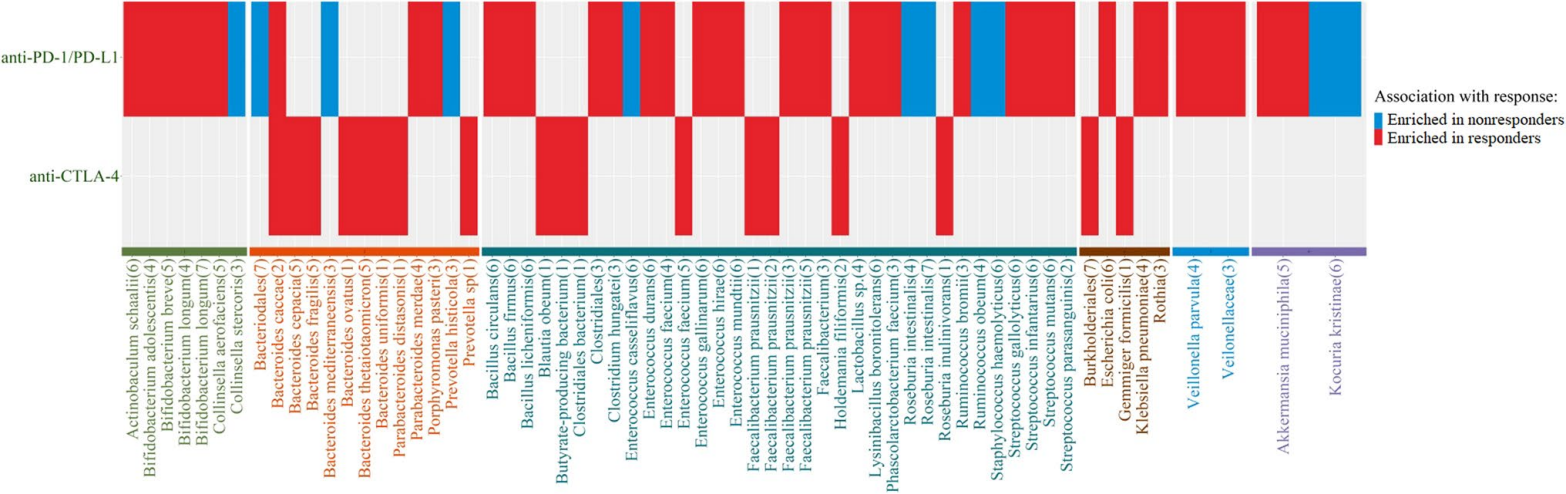
Differences in the enrichment of intestinal microbiota across different immune checkpoints. Various affinity effects of different microbial groups on ICIs are outlined. Intestinal microbiota associated with a positive response is marked in red and include enriched in responders such as *Bifidobacterium* species, *Akkermansia muciniophila*, and *Faecalibacterium prausnitzii*, among others. Conversely, those associated with negative responses are marked with blue, including those enriched in non-responders such as *Collinsella stercoris*, Bacteriodales, and *Roseburia intestinalis* among others. This evidence is expected to guide the development of novel cancer immunotherapies. Numbers in brackets denote sources of the references. (1) [56]; (2) [57]; (3) [58]; (4) [59]; (5) [60]; (6) [61]; (7) [62]

In recent years, the use of intestinal microbiota has increasingly become a focus of cancer immunotherapy [7]. These communities of microorganisms not only participate in the immune regulation of different periods but can also regulate the tumor microenvironment and affect the efficacy of ICIs. However, their underlying mechanism of action in cancer immunotherapy remains unclear, necessitating in-depth investigation. Clinical researches unraveling the mechanism of microbiota action in cancer immunotherapy can guide the development of effective individualized clinical treatment approaches. Here, we have summarized the role of intestinal microbiota in clinical trials related to cancer immunotherapy (Table 1) and generated a schematic representation of the degree of correlation between intestinal microbiota and immune checkpoints (PD-1/PD-L1 and CTLA-4) (Fig. 2).

**Table 1:** Trials of microbial-related immunotherapy

NCT number	Title	Status	Conditions	Interventions	Phases	
NCT04638751	ARGONAUT: Stool and Blood Sample Bank for Cancer Patients	Recruiting	Non-Small Cell Lung Cancer Colorectal Cancer Triple Negative Breast Cancer Pancreas Cancer	Drug: Immunotherapy Drug: Chemotherapeutic Agent		
NCT04636775	Fecal Microbiota Transplant (FMT) in Melanoma Patients	Recruiting	Microbiome in Immunotherapy naïve NSCLC Patients Receiving PD-1/L1 Blockade	Diagnostic Test: Microbiome		
NCT04579978	Tumor Immunotherapy and Microbiome Analysis	Recruiting	Advanced Solid Tumor			
NCT04552418	Intestinal Microbiome Modification With Resistant Starch in Patients Treated With Dual Immune-Checkpoint Inhibitors	Not yet recruiting	Solid Tumor	Dietary Supplement: Potato starch	Early Phase 1	
NCT04363983	Interaction Between Host, Microenvironment, and Immunity on Gastrointestinal Neoplasms	Not yet recruiting	Gastrointestinal Neoplasms	Biological: Blood sampling Procedure: Liver biopsy Biological: Stool collect		
NCT04264975	Utilization of Microbiome as Biomarkers and Therapeutics in Immuno-Oncology	Recruiting	Solid Carcinoma	Procedure: fecal microbiota transplantation		
NCT04189679	Identification of a Predictive Metabolic Signature of Response to Immune-Checkpoint Inhibitors in NSCLC	Recruiting	Non-Small Cell Lung Cancer	Other: Immune signature in serum associated with the metabolic signature Genetic: Meta-genomic signature of intestinal flora		
NCT04163289	Preventing Toxicity in Renal Cancer Patients Treated With Immunotherapy Using Fecal Microbiota Transplantation	Recruiting	Renal Cell Carcinoma	Drug: Fecal Microbiota Transplantation	Phase 1	
NCT04130763	Fecal Microbiota Transplant (FMT) Capsule for Improving the Efficacy of Anti-PD-1	Recruiting	Gastrointestinal System Cancer	Biological: FMT capsule	Phase 1	
NCT04107168	Microbiome Immunotherapy Toxicity and Response Evaluation	Recruiting	Melanoma Renal Cancer Lung Cancer	Drug: Nivolumab Drug: Pembrolizumab Drug: Ipilimumab Drug: Durvalumab Drug: Tremelimumab Drug: Atezolizumab Drug: Bevacizumab		
NCT04056026	A Single Dose FMT Infusion as an Adjunct to Keytruda for Metastatic Mesothelioma	Completed	Mesothelioma	Biological: Fecal Microbiota Transplant	Early Phase 1	
NCT04054908	Gut Microbiome in Colorectal Cancer	Recruiting	Gastrointestinal Microbiome Neoplasm, Colorectal			
NCT04038619	Fecal Microbiota Transplantation in Treating Immune-Checkpoint Inhibitor Induced-Diarrhea or Colitis in Genitourinary Cancer Patients	Not yet recruiting	Colitis Diarrhea Malignant Genitourinary System Neoplasm	Procedure: Fecal Microbiota Transplantation Drug: Loperamide	Phase 1	
NCT03891979	Gut Microbiome Modulation to Enable Efficacy of Checkpoint-based Immunotherapy in Pancreatic Adenocarcinoma	Withdrawn	Pancreatic Cancer	Drug: Pembrolizumab Drug: Ciprofloxacin 500 mg PO BID days 1–29 Drug: Metronidazole 500 mg PO TID days 1–29	Phase 4	
NCT03772899	Fecal Microbial Transplantation in Combination With Immunotherapy in Melanoma Patients (MIMic)	Recruiting	Melanoma	Drug: Fecal Microbial Transplantation	Phase 1	
NCT03688347	Microbiome in Lung Cancer and Other Malignancies	Active, not recruiting	Lung Cancer Cancer Malignancy	Procedure: Nasal Swab Procedure: Oral Swab Other: Stool Collection Genetic: Microbiome analysis Genetic: DNA Banking Procedure: Skin Swab		
NCT03686202	Feasibility Study of Microbial Ecosystem Therapeutics (MET-4) to Evaluate Effects of Fecal Microbiome in Patients on Immunotherapy	Recruiting	All Solid Tumors	Biological: MET-4	Early Phase 1	
NCT03643289	Predicting Response to Immunotherapy for Melanoma With Gut Microbiome and Metabolomics	Recruiting	Melanoma (Skin)			
NCT03557749	Monitoring of Immune and Microbial Reconstitution in (HCT) and Novel Immunotherapies	Recruiting	Immune and Microbial Reconstitution Systemic Viral Infection Acute-graft-versus-host Disease Chronic Graft-versus-host-disease Recurrent Malignancy Cytokine Release Syndrome Allogenic Related Donors Cell Therapy/Immunotherapy Patients	Diagnostic Test: Blood Sample Diagnostic Test: Stool Sample Diagnostic Test: Urine Sample Diagnostic Test: Bronchoalveolar Lavage (BAL) fluid Diagnostic Test: Gastrointestinal biopsy × 2–4 Diagnostic Test: Skin biopsy Diagnostic Test: Skin, mouth, ocular swab Diagnostic Test: Apheresis Product Diagnostic Test: Final cellular product		
NCT03383107	Effect of Radiotherapy Variables on Circulating Effectors of Immune Response and Local Microbiome	Recruiting	Breast Cancer Prostate Cancer			
NCT03353402	Fecal Microbiota Transplantation (FMT) in Metastatic Melanoma Patients Who Failed Immunotherapy	Recruiting	Melanoma Stage IV Unresectable Stage III Melanoma	Procedure: Fecal Microbiota Transplant (FMT)	Phase 1	
NCT02960282	Gut Microbiome in Fecal Samples From Patients With Metastatic Cancer Undergoing Chemotherapy or Immunotherapy Melanoma Patients Who Failed Immunotherapy	Recruiting	Metastatic Carcinoma Stage IV Colorectal Cancer Stage IVA Colorectal Cancer Stage IVB Colorectal Cancer	Procedure: Biospecimen Collection Other: Laboratory Biomarker Analysis

Gopalakrishnan et al. has found that *Bifidobacteria* could enhance anti-tumor immunity and improve the efficacy of anti-PD-L1 immunotherapy against melanoma in mouse models [58]. However, the researchers were not sure whether the symbiotic microbiota interfered with the therapeutic activity of ICIs on the host's immune response. Consequently, Sivan et al. tested this association by assessing the growth rate of melanoma from two different sources (JAX/TAC) harboring carrying different intestinal microbiota, and genetically similar mice. Their results showed that JAX and TAC mice exhibited different anti-tumor effects, among which JAX mice manifested a stronger tumor-specific T cell response and more CD8^+^ T cells in the tumor [63]. However, these differences disappeared after the application of fecal bacteria transplantation technology or co-raising, suggesting that the intestinal microbiota significantly impacted the host's anti-tumor response [63]. Meanwhile, PD-L1 inhibitors are more effective in JAX mice. In TAC mice, the inhibitory effect on tumor growth was comparable to that of PD-L1 inhibitors, although this was only after transplantation of fecal microbiota from JAX mice [63]. In a parallel experiment, in the context of PD-1 blockade, researchers compared the growth of genetically similar mice with subcutaneous melanoma to those containing different symbiotic microbiota and found significantly different tumor growth rates between the groups [64]. In addition, analysis of the intestinal microbiome showed that *Bifidobacteria* could induce activation of dendritic cells [65], improve the effect of tumor-specific CD8^+^ T cells, enhance anti-tumor immunity and inhibition of PD-L1 [66]. Overall, these findings affirmed the important role played by intestinal microbiota in cancer immunotherapy. Moreover, intestinal microbiota has been demonstrated to play a key role in human health and disease, particularly in the local and systemic regulation of host immunity. Conversely, the immune microenvironment influences the function of intestinal microbiota to varying degrees. These changes are attributed to a variety of factors, including diet, sleep cycle, exercise, and drugs that have a direct or indirect interference with the composition and metabolic functions of the intestinal microbiota. Therefore, these parameters may also exert effects on the properties and efficacy of the intestinal microbiota and ICIs in the same or heterogeneous tumors.

Coincidentally, recent studies have focused on the impact of intestinal microbiota on immunotherapy, and found that through regulation of intestinal microbiota, they significantly improve immunotherapeutic response. Different microbiota responds differently to ICIs treatment. We have summarized the regulatory effect of intestinal microbiota in immunotherapy in Table 2.

**Table 2:** Regulation of intestinal microbiota in cancer immunotherapy

Microbiota (or products) involved	Immune regulations	Effects on response/toxicity	Impact on cancer immunotherapy	Cancers	References
*Alistipes putredinis*	Increasing unique memory CD8^+^ T cells and NK cells in periphery	Improving immunotherapy response	Enhancing PD-1 blockade effect	NSCLC RCC	[60]
*Akkermansia muciniphila*	Increasing CXCR3^+^CCR9^+^CD4^+^ T cells Enhancing the expression of IL-12 and the function of DCs	Improving immunotherapy response	Enhancing PD-1 blockade effect	NSCLC RCC	[60]
*Bacteroides spp.*	Up-regulating the system's MDSC and Tregs Causing systemic inflammatory response through the TLR-NF inflammatory pathway Reducing the secretion of IL-12 and the production of DCs	Diminishing the risk of ICIs-induced colitis	Impeding PD-1 blockade effect Impeding CTLA-4 blockade effect	MM	[56]
*Bacteriodes fragilis*	Activating Th1 cells Promoting Foxp3^+^ Tregs proliferation Promoting maturation of DCs	Promoting tumor control Preserving intestinal integrity	Enhancing CTLA-4 blockade effect	MM NSCLC	[67]
*Bifidobacterium spp.*	Promoting maturation of DCs Increasing the activity of lymphocytes Up-regulating the expression of IFN-γ Increasing pro-inflammatory cytokine Priming tumor-specific CD8^+^ T cells	Enhancing the activity of CD8^+^ T cells in TME Inhibiting the growth of melanoma	Enhancing PD-1 blockade effect	MM	[59] [63]
*Enterococcus faecium*	Augmenting T cell responses	Improving immunotherapy response	Enhancing PD-1 blockade effect	MM	[58]
*Escherichia* *Clostridium*	Promoting differentiation of Tregs Suppressing the invasion of inflammation	Enhancing systemic tumor immunity	Enhancing CTLA-4 blockade effect	MM	[68]
*Faecalibacterium. spp.*	Inducing the proliferation of CD4^+^ or CD8^+^ T cells Enhancing the production and differentiation of Tregs Up-regulating ICOS expression of T cells	Diminishing the risk of ICIs-induced colitis and improving immunotherapy response	Enhancing PD-1 blockade effect Enhancing CTLA-4 blockade effect	MM	[56]
*Ruminococcaceae spp.*	Increasing antigen presentation Improving effector T cell function in TME Inducing IFN-γ CD8^+^ T cells	Enhancing response to resistant patients	Impeding PD-1 blockade effect	MM	[59]
Microbial-derived SCFAs (butyrate, propionate)	Promoting the differentiation of Tregs	Increasing the acetylation level of histone H3 in the Foxp3 promoter region	Enhancing CTLA-4 blockade effect	CRC	[69]

Researchers have also performed quantitative metagenomics using shotgun sequencing on stool samples obtained from NSCLC and RCC patients and found that the composition of the intestinal microbiota of responders (R) to anti-PD-1 treatment differs significantly from that of non-responders (NR) [61]. Specifically, intestinal microbiota from responders predominantly comprises *Firmicutes*, as well as different other bacterial genera, such as *Akkermansia muciniphila* and *Alitipes *[60]. Further analysis of the progression-free survival (PFS) in 53 patients in a validation cohort revealed that *Akkermansia muciniphila* was enriched in patients with PFS over 3 months, while the microbiota was lacking in patients with PFS less than 3 months [60, 61]. Furthermore, the researchers found that *Akkermansia muciniphila* accounted for the majority of the detected bacteria in the stool of patients with positive therapeutic effects, after PD-1 blockade treatment [61]. In a prospective study involving 39 patients with metastatic melanoma treated with ICIs, patients with significant responses had a large number of *Bacteroides caccae* [57]. In addition, the researchers compared the gut microbiome composition of R and NR to anti-PD-1 treatment and found that the former group had significantly higher diversity and abundance of *Clostridiaceae, Rumenococcus,* and *Faecalis* relative to the former [59]. Notably, improvements in systemic and anti-tumor immunity were also observed in R with good intestinal microbiota [70]. Another group of patients with metastatic melanoma also exhibited a significant association between response to ICIs and the intestinal microbiota. Specifically, responders manifested a large number of *Bifidobacterium longum*, *Collinsella aerofaciens,* and *Enterococcus faecalis* than non-responders [59]. Collectively, these findings demonstrate that homeostasis of the intestinal microbiota potentially promotes the blocking effect of PD-1/PD-L1 and CTLA-4, improves response to ICIs, and promotes tumor control.

Based on a consensus among the above-mentioned studies, it is evident that patients with good intestinal microbiota will have improved T cell function in the tumor microenvironment through the intestinal microbiota, which subsequently enhances the efficacy of ICIs. Conversely, patients with poor gut microbiota exhibit a weakened anti-tumor immunity by limiting antigen presentation. Based on this, it is clear that intestinal microbiota guides the direction of individualized immunotherapy for tumor patients, hence it is a promising approach for reducing resistance to ICIs through alteration of the intestinal micro-ecosystem. In the future, we hypothesize that Fecal Microbiota Transplant (FMT) may become a new method for interfering with the intestinal microbiota. Table 1 shows multiple clinical trials (NCT04636775, NCT04130763, NCT04038619, NCT03772899), and the safety and feasibility researches of FMT combined with cancer immunotherapy (pembrolizumab/nivolumab) are also under clinical trials. Although FMT is only a prototype in the exploration of cancer immunotherapy, it is undeniable that it is a milestone. These clinical trials are expected to further promote research targeting the application of FMT in cancer immunotherapy and guide the development of effective clinical anticancer therapies.

## Antibiotic therapy negatively regulates ICIs during cancer immunotherapy

Epidemiological researches have revealed the harmful effects of antibiotics on immunotherapy. For example, chronic antibiotic therapy can cause disturbances in intestinal microbiota, potentially reducing the associated ICI benefits [71]. A study found that strong dependence on antibiotic exposure time doubled the risk of immunotherapy and shortened patient survival by 20 months [72]. However, this had nothing to do with established prognostic factors and the use of glucocorticoids [73]. Moreover, previous pre-clinical evidence has revealed reduced response to ICIs in tumor-bearing mice following antibiotic pretreatment [74], but the underlying mechanisms of antibiotics and immunotherapy remain unclear. Currently, results from clinical trials and conventional treatment therapies have associated antibiotic exposure with low overall survival and response rates of patients undergoing immunotherapy [75, 76]. Several studies have demonstrated a negative correlation between antibiotic exposure with outcomes in patients and advanced solid cancer who received ICIs [77–81]. In another study, Tinsley et al. performed a retrospective study of antibiotic exposures in patients treated with ICIs during the first two and six weeks of treatment, of which 92 patients (32%) received antibiotic treatment, and found that cumulative antibiotic exposure also limited efficacy of ICIs. Univariate analysis showed that progression-free survival (PFS) was associated with antibiotic exposure, production status, and comorbidities. Similarly, overall survival (OS) was correlated with the above three factors, clinical trial enrollment, and more than three metastatic sites [77]. In the experiment, where patients were divided into three groups, namely no antibiotic treatment, a single course of antibiotic treatment (not more than 7 days), and cumulative antibiotic treatment (more than 7 days), the authors found that patients receiving cumulative antibiotic treatment exhibited significantly shorter PFS and OS. However, the single-course antibiotic treatment group had an inferior effect on ICIs, and it was not significantly related to the trend of PFS and OS reduction [77]. Among them, patients in the antibiotic-free treatment group exhibited a longer median survival time than those in the single course/cumulative antibiotic treatment group [77]. These findings indicate that antibiotic exposure in advanced tumor patients with ICIs is associated with poor patient prognosis.

Similarly, Ahmed et al. also conducted a retrospective cohort study involving 60 patients who received ICIs and found significantly lower response rates (RR) as well as PFS and OS in patients who received antibiotic therapy 2 weeks before and after the first immunotherapy [82]. Interestingly, a phase I clinical trial revealed that the use of antibiotics, 1 month before immunotherapy could reduce OS, but a similar therapy within 1–2 months after the start did not affect PFS and OS [80]. However, two small retrospective studies failed to reach a consensus regarding the link between antibiotic therapy and the efficacy of ICIs, although their study populations were limited to NSCLC patients who received nivolumab treatment [77, 83]. In the first prospective study in this field, Pinato et al. found that the use of antibiotic therapy before ICI treatment was associated with poor OS (*P* < 0.001), and was strongly correlated with the initial refractory of ICIs (*P* < 0.001). In contrast, the above phenomenon was not observed in patients treated with antibiotics after ICIs treatment (*P* = 0.76). Multivariate analysis showed that in all types of tumors, the negative effects of previous antibiotic treatments were independent of tumor location and functional status [72]. Furthermore, current evidence suggests the existence of a surprise interaction between antibiotic exposure and prognosis in patients with ICIs. In the context of immunotherapy, [77] found that the use of multiple or long-term antibiotics seems to be more disadvantageous than single or short-term treatments. However, their study was limited by its retrospective design, as well as recruitment of patients from a single-center, which may have led to biases in patient selection and analysis. In addition, their study did not evaluate the effect of antibiotic exposure time on ICI-based treatment.

Antibiotics potentially are associated with invisible side effects on the intestinal microbiota, which consequently impact the development and function of immune cells. Following the inhibition of intestinal probiotics by antibiotics, the number and performance of naïve cells, Th1/Th2 cells, and Tregs change, and the cells become more susceptible to infection and sepsis [84]. Features associated with immune dysfunction, among them, low expression of antimicrobial peptides, decreased IgA secretion, and downregulation of IFN-γ, have been described in patients subjected to antibiotic treatment [84]. Because antibiotics influence the intestinal microbiota, they also interfere with the efficacy of ICIs. With regards to the impact on clinical outcomes, more convincing data is expected from the evaluation of patients who receive antibiotic intervention during immunotherapy. Unfortunately, studies that have evaluated the relationship between microbiota, antibiotics, and ICIs are mainly retrospective, hence cannot accurately distinguish the effects of different antibiotic types. Given the limited number of clinical trials evaluating the efficacy of antibiotics in immunotherapy, any speculation regarding the association of antibiotics with ICIs can only be deduced from clinical evidence related to microbes or antibiotics and tumor immunotherapy. For instance, a recent study on the effects of antibiotics on ICIs showed progress, including MM (*n* = 201), NSCLC (*n* = 56), and RCC (*n* = 46) [77]. Among them, nearly one-third of the patients (*n* = 94) received antibiotics (β-lactams and macrolides) before or during treatment with ICIs. Multivariate analysis revealed that this group of patients exhibited significantly lower PFS and OS relative to those who did not receive antibiotics. Moreover, patients treated with antibiotics (before ICIs) exhibited significantly shorter PFS and OS than those who took antibiotics after ICIs. Similarly, Routy et al. analyzed 249 immunotherapy cases, including NSCLC (*n* = 140), RCC (*n* = 67) and urothelial carcinoma (*n* = 42), with 69 cases receiving antibiotic treatment (β-lactams, fluoroquinolones or macrolides) prior to ICIs administration [60]. Their results demonstrated that patients in the antibiotics group exhibited significantly shorter PFS and OS. Moreover, Huang et al. conducted a meta-analysis of the interaction between antibiotics with ICIs, and found a significant correlation between antibiotics with unfavorable PFS and OS [85]. This analysis is applicable to different types of tumors, and has nothing to do with the time of antibiotic use.

To evaluate the relationship between antibiotics and cancer response to ICIs, we compiled a summary of relevant researches over the recent years (Table 3). Summarily, both PFS and OS are expected to decrease within a few weeks of receiving ICIs for NSCLS and melanoma. Since these clinical reports are observational, it is easy to confuse their outcomes with host factors associated with a high risk of infection and poor prognosis. However, results from multivariate analysis indicate that most of the outcomes remain statistically significant. In addition, these clinical observations are supported by data, indicating that the causal relationship between the use of antibiotics and the failure of ICIs treatment may be the destruction of the microbiota, hence play a key role in inhibiting a host's immune response to tumor cells.

**Table 3:** Retrospective studies on antibiotic exposure and clinical efficacy of patients receiving ICIs treatment

Malignancy	ICIs (anti-)	Antibiotics	Duration	PFS	OS	PD	Notes	References
				Univariate analysis				
NSCLC	PD(L)-1	–	− 3 m	–	NR	–	–	[91]
NSCLC	PD(L)-1	β-lactam Sulfonamids Quinolones	− 1 m	↓	↓	NR	On multivariate analysis, OS *p* = 0.19	[79]
NSCLC	PD(L)-1	β-lactam Fluoroquinolones Carbapenems	− 1 ~ 1 m	–	–	NR	73% patients in the antibiotic group received penicillin	[81]
NSCLC RCC	PD(L)-1 CTLA-4	β-lactam Fluoroquinolones	− 1 ~ 2 m	↓	↓	–	On multivariate analysis, OS *p* < 0.05, PFS *p* = 0.17 p-value non-significant when time extended to − 2 m	[83]
				↓	↓	↑	On multivariate analysis, PFS *p* < 0.05; OS *p* = 0.11	
Melanoma	PD(L)-1 CTLA-4	β-lactam	− 1 m	↓	–	↑	On multivariate analysis, PFS *p* < 0.05	[92]
NSCLC RCC UC	PD(L)-1	β-lactam Fluoroquinolones Macrolides	− 2 ~ 1 m	–	↓	NR	–	[61]
				↓	–	NR		
				↓	↓	NR	On multivariate analysis, OS *p* = 0.098	
Melanoma NSCLC RCC	PD(L)-1 CTLA-4	β-lactam Macrolides	− 2 ~ 6w	↓	↓	NR	On multivariate analysis, OS and PFS *p* < 0.05 Worse OS and PFS with > 1 antibiotic courses	[77]
RCC Melanoma NSCLC	PD(L)-1 CTLA-4	Quinolones β-lactam Tetracyclines	− 1 ~ 2 m	–	↓	–	–	[80]
NSCLC RCC UC Melanoma	PD(L)-1	β-lactam Quinolone Vancomycin Tetracyclines Macrolides	− 2 ~ 2w	↓	↓	↑	On multivariate analysis, PFS *p* < 0.05 Narrow-spectrum (anti-Gram) antibiotics had no effect	[82]
NSCLC	PD(L)-1	–	− 1 ~ 3 m	↓	↓	NR	On multivariate analysis, PFS *P* < 0.05; OS *p* < 0.0001	[93]
NSCLC Melanoma	PD(L)-1 CTLA-4	–	− 4 ~ 4w	↓	↓	NR	On multivariate analysis, PFS *P* = 0.007; OS *p* = 0.02	[94]
NSCLC Melanoma	PD(L)-1 CTLA-4	β-lactam	− 1 m ~ cessation	↓	↓	NR	On multivariate analysis, PFS *P* = 0.049; OS *P* = 0.001	[72]
NSCLC RCC	PD(L)-1	Fluoroquinolones Macrolides Tetracyclines Cephalosporins Penicillins	− 4 ~ 6w	↓	↓	NR	–	[76]
							On multivariate analysis, OS *P* = 0.37	
Melanoma	PD(L)-1 CTLA-4	Cephalosporins Penicillins Fluoroquinolones	− 3 m ~ infusion	–	↓	NR	On multivariate analysis, penicillins, cephalosporins and fluoroquinolones were associated with worse OS	[95]

In some tumors, to eliminate the imbalance of the microbiota, antibiotics are used to regulate the immunosuppression of cancer patients. The intestinal microbiota in some patients with pancreatic cancer can promote immunosuppression and tumor immune escape by inducing the proliferation of Foxp3^+^ Tregs [86]. In this case, appropriate antibiotics may reduce the abundance of such microbiota, thereby improving tumor control. Pushalkar et al. found that microbial ablation can induce immunogenic reprogramming of the tumor microenvironment and increase the expression of PD-1 on T cells [87]. This suggests that the combination of antibiotics and ICIs may be an attractive cancer treatment strategy. Unfortunately, there is still a lack of research on how antibiotics can eliminate or regulate immunosuppression in cancer patients. And it is necessary to conduct prospective studies to identify tumor-specific microbial characteristics, which may be beneficial to early diagnosis and subsequent treatment.

**Fig. 3:**
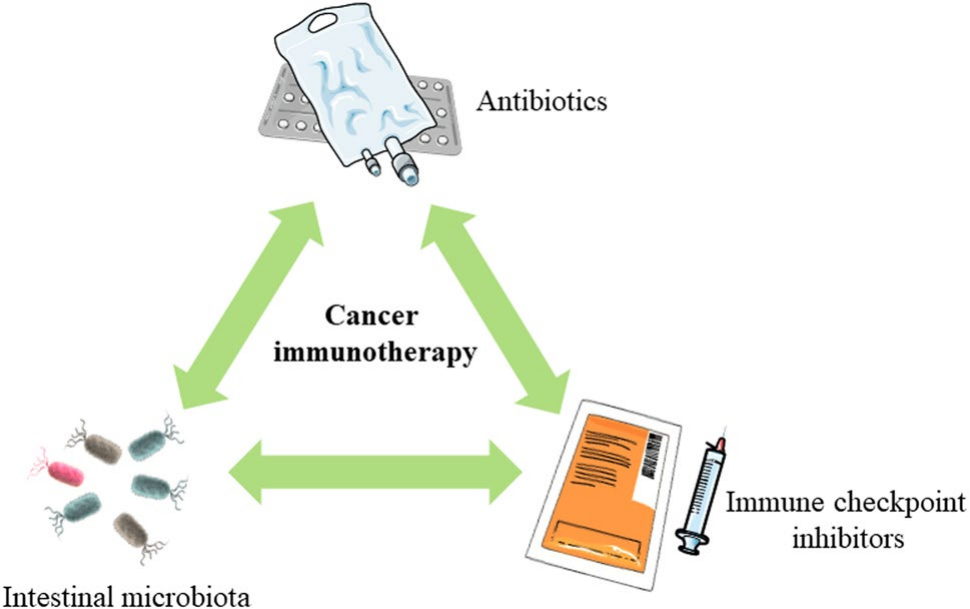
A new cancer biology triangle formed by intestinal microbiota, ICIs and antibiotics

We believe more large-scale researches are needed to provide detailed statistical stratification of cancer and immunotherapy types. Moreover, it is necessary to classify the duration of ICIs and the course of different antibiotics. Considering the potential for antibiotics to produce long-term adverse effects during ICI-based treatments, it is imperative to design effective management interventions for cancer patients receiving ICIs treatment or considering the use of empirical antimicrobials for target populations taking antibiotics. Apart from analyzing the effect of antibiotics on ICIs, it is important to investigate their effect on microbiota. Most studies have emphasized the importance of a healthy intestinal microbiota for a good response to ICIs. Unfortunately, insufficient data or antibiotic exposure has made it difficult to detect the effects of intestinal microbiota after interference and clinically meaningful results [88]. A comprehensive understanding of the role of intestinal microbiota during immunotherapy will guide their effective use, including evaluation of their characteristics before treatment, the possibility of predicting response to treatment, as well as informing meaningful making of therapeutic decisions. This intervention has previously shown promise in pre-clinical mouse models of solid cancer where sterile or antibiotic-treated mice exhibited impaired ICIs response, relative to controls [89]. However, the efficacy of ICIs can be restored through interventions, such as cohabitation with mice with immune responses, microbial supplementation, or FMT [90]. Intestinal microbiota, ICIs and antibiotics are likely to become a new cancer biology triangle (Fig. 3). This will generate new insights to guide future improvement of immunotherapies.

## Conclusion

Human intestinal microbiota participates in host immune response and regulates multiple steps of the tumor immune cycle, including antigen presentation, T cell initiation, and activation. Exploring the immune mechanism underlying intestinal microbiota action in the body is not only beneficial to patient prognosis but also plays a key role in the future development of microbiota-based cancer immunotherapies. In addition, the use of antibiotics has now been shown to be a dynamic factor affecting the efficacy of ICIs. Based on the studies reviewed herein, it is evident that the use of antibiotics can shorten the PFS and OS of patients within 2–3 months before or after the start of immunotherapy. Recognizing changes in the microbiota will facilitate diversification and individualization of clinical treatments, such as the use of selective antibiotics, intervention of prebiotics or probiotics, dietary intervention, or FMT, among others. To date, researchers have focused on clinical induction of the body's immune system through regulation of intestinal microbiota to improve the efficacy of immunotherapy, and overcome resistance and adverse reactions during the process. In the future, exploring the feasibility of manipulating intestinal microbiota is expected to generate vital insights that will positively impact cancer immunotherapy.

## Abbreviations

APCs: Antigen-presenting cells
CTLA-4: Cytotoxic T lymphocyte antigen 4
FMT: Fecal Microbiota Transplant
ICIs: Immune checkpoint inhibitors
ITSM: Immunoreceptor tyrosine-based switch motif
ITIM: Immunoreceptor tyrosine-based inhibitory motif
NSCLC: Non-small cell lung cancer
OS: Overall survival
PD-1/PD-L1: Programmed death receptor 1/programmed death-ligand 1
PFS: Progression-free survival
RR: Response rate
TPS: Tumor proportional score

## References

[1] Qiu Q, Lin Y, Ma Y, Li X, Liang J, Chen Z, Liu K, Huang Y, Luo H, Huang R, Luo L (2020). Exploring the emerging role of the gut microbiota and tumor microenvironment in cancer immunotherapy. Front Immunol.

[2] Vujkovic-Cvijin I, Sklar J, Jiang L, Natarajan L, Knight R, Belkaid Y (2020). Host variables confound gut microbiota studies of human disease. Nature.

[3] Zhang X, Pan Z (2020). Influence of microbiota on immunity and immunotherapy for gastric and esophageal cancers. Gastroenterol Rep (Oxf).

[4] Ge Y, Wang X, Guo Y, Yan J, Abuduwaili A, Aximujiang K, Yan J, Wu M (2021). Gut microbiota influence tumor development and Alter interactions with the human immune system. J Exp Clin Cancer Res.

[5] Longhi G, van Sinderen D, Ventura M, Turroni F (2020). Microbiota and cancer: the emerging beneficial role of Bifidobacteria in cancer immunotherapy. Front Microbiol.

[6] Fishman JA, Hogan JI, Maus MV (2019). Inflammatory and infectious syndromes associated with cancer immunotherapies. Clin Infect Dis.

[7] Dai Z, Zhang J, Wu Q, Fang H, Shi C, Li Z, Lin C, Tang D, Wang D (2020). Intestinal microbiota: a new force in cancer immunotherapy. Cell Commun Signal.

[8] Feng M, Xiong G, Cao Z, Yang G, Zheng S, Song X, You L, Zheng L, Zhang T, Zhao Y (2017). PD-1/PD-L1 and immunotherapy for pancreatic cancer. Cancer Lett.

[9] Callea M, Pedica F, Doglioni C (2016). Programmed death 1 (PD-1) and its ligand (PD-L1) as a new frontier in cancer Immunotherapy and challenges for the Pathologist: state of the art. Pathologica.

[10] Zheng P, Zhou Z (2015). Human cancer immunotherapy with PD-1/PD-L1 blockade. Biomark Cancer.

[11] Leung J, Suh WK (2014). The CD28-B7 family in anti-tumor immunity: emerging concepts in cancer immunotherapy. Immune Netw.

[12] Rota G, Niogret C, Dang AT, Barros CR, Fonta NP, Alfei F, Morgado L, Zehn D, Birchmeier W, Vivier E, Guarda G (2018). Shp-2 is dispensable for establishing T Cell exhaustion and for PD-1 signaling in vivo. Cell Rep.

[13] Li J, Jie HB, Lei Y, Gildener-Leapman N, Trivedi S, Green T, Kane LP, Ferris RL (2015). PD-1/SHP-2 inhibits Tc1/Th1 phenotypic responses and the activation of T cells in the tumor microenvironment. Cancer Res.

[14] Xie J, Han X, Zhao C, Canonigo-Balancio AJ, Yates  JR, Li Y, Lillemeier BF, Altman A (2019). Phosphotyrosine-dependent interaction between the kinases PKCtheta and Zap70 promotes proximal TCR signaling. Sci Signal.

[15] Barclay J, Creswell J, Leon J (2018). Cancer immunotherapy and the PD-1/PD-L1 checkpoint pathway. Arch Esp Urol.

[16] Lo WL, Shah NH, Ahsan N, Horkova V, Stepanek O, Salomon AR, Kuriyan J, Weiss A (2018). Lck promotes Zap70-dependent LAT phosphorylation by bridging Zap70 to LAT. Nat Immunol.

[17] Bommhardt U, Schraven B, Simeoni L (2019). Beyond TCR signaling: emerging functions of Lck in cancer and immunotherapy. Int J Mol Sci.

[18] Kalathur M, Toso A, Chen J, Revandkar A, Danzer-Baltzer C, Guccini I, Alajati A, Sarti M, Pinton S, Brambilla L (2015). A chemogenomic screening identifies CK2 as a target for pro-senescence therapy in PTEN-deficient tumours. Nat Commun.

[19] Borgo C, Ruzzene M (2019). Role of protein kinase CK2 in antitumor drug resistance. J Exp Clin Cancer Res.

[20] Ohaegbulam KC, Assal A, Lazar-Molnar E, Yao Y, Zang X (2015). Human cancer immunotherapy with antibodies to the PD-1 and PD-L1 pathway. Trends Mol Med.

[21] Spranger S, Bao R, Gajewski TF (2015). Melanoma-intrinsic beta-catenin signalling prevents anti-tumour immunity. Nature.

[22] Peng W, Chen JQ, Liu C, Malu S, Creasy C, Tetzlaff MT, Xu C, McKenzie JA, Zhang C, Liang X (2016). Loss of PTEN promotes resistance to T cell-mediated immunotherapy. Cancer Discov.

[23] Zlotorynski E (2017). Non-coding RNA: the cancer link(RNA) between PIP3 and AKT. Nat Rev Mol Cell Biol.

[24] Narayanankutty A (2019). PI3K/ Akt/ mTOR pathway as a therapeutic target for colorectal cancer: a review of preclinical and clinical evidence. Curr Drug Targets.

[25] Shimizu T, Tolcher AW, Papadopoulos KP, Beeram M, Rasco DW, Smith LS, Gunn S, Smetzer L, Mays TA, Kaiser B (2012). The clinical effect of the dual-targeting strategy involving PI3K/AKT/mTOR and RAS/MEK/ERK pathways in patients with advanced cancer. Clin Cancer Res.

[26] Loi S, Dushyanthen S, Beavis PA, Salgado R, Denkert C, Savas P, Combs S, Rimm DL, Giltnane JM, Estrada MV (2019). Correction: RAS/MAPK activation is associated with reduced tumor-infiltrating lymphocytes in triple-negative breast cancer: therapeutic cooperation between MEK and PD-1/PD-L1 immune checkpoint inhibitors. Clin Cancer Res.

[27] Patsoukis N, Brown J, Petkova V, Liu F, Li L, Boussiotis VA (2012). Selective effects of PD-1 on Akt and Ras pathways regulate molecular components of the cell cycle and inhibit T cell proliferation. Sci Signal.

[28] Wu X, Gu Z, Chen Y, Chen B, Chen W, Weng L, Liu X (2019). Application of PD-1 blockade in cancer immunotherapy. Comput Struct Biotechnol J.

[29] Chretien S, Zerdes I, Bergh J, Matikas A, Foukakis T (2019). Beyond PD-1/PD-L1 inhibition: what the future holds for breast cancer immunotherapy. Cancers (Basel).

[30] Rotte A (2019). Combination of CTLA-4 and PD-1 blockers for treatment of cancer. J Exp Clin Cancer Res.

[31] Callahan MK, Wolchok JD (2013). At the bedside: CTLA-4- and PD-1-blocking antibodies in cancer immunotherapy. J Leukoc Biol.

[32] Ganesan A, Moon TC, Barakat KH (2018). Revealing the atomistic details behind the binding of B7–1 to CD28 and CTLA-4: a comprehensive protein-protein modelling study. Biochim Biophys Acta Gen Subj.

[33] Buchbinder EI, Desai A (2016). CTLA-4 and PD-1 pathways: similarities, differences, and implications of their inhibition. Am J Clin Oncol.

[34] Masteller EL, Chuang E, Mullen AC, Reiner SL, Thompson CB (2000). Structural analysis of CTLA-4 function in vivo. J Immunol.

[35] Spranger S, Koblish HK, Horton B, Scherle PA, Newton R, Gajewski TF (2014). Mechanism of tumor rejection with doublets of CTLA-4, PD-1/PD-L1, or IDO blockade involves restored IL-2 production and proliferation of CD8(+) T cells directly within the tumor microenvironment. J Immunother Cancer.

[36] Torrealba N, Rodriguez-Berriguete G, Fraile B, Olmedilla G, Martinez-Onsurbe P, Sanchez-Chapado M, Paniagua R, Royuela M (2018). PI3K pathway and Bcl-2 family. Clinicopathological features in prostate cancer. Aging Male.

[37] Tanaka A, Sakaguchi S (2019). Targeting Treg cells in cancer immunotherapy. Eur J Immunol.

[38] Ohue Y, Nishikawa H (2019). Regulatory T (Treg) cells in cancer: can Treg cells be a new therapeutic target?. Cancer Sci.

[39] Topalian SL, Hodi FS, Brahmer JR, Gettinger SN, Smith DC, McDermott DF, Powderly JD, Carvajal RD, Sosman JA, Atkins MB (2012). Safety, activity, and immune correlates of anti-PD-1 antibody in cancer. N Engl J Med.

[40] Robert C, Long GV, Brady B, Dutriaux C, Maio M, Mortier L, Hassel JC, Rutkowski P, McNeil C, Kalinka-Warzocha E (2015). Nivolumab in previously untreated melanoma without BRAF mutation. N Engl J Med.

[41] Mok TSK, Wu YL, Kudaba I, Kowalski DM, Cho BC, Turna HZ, Castro G, Srimuninnimit V, Laktionov KK, Bondarenko I (2019). Pembrolizumab versus chemotherapy for previously untreated, PD-L1-expressing, locally advanced or metastatic non-small-cell lung cancer (KEYNOTE-042): a randomised, open-label, controlled, phase 3 trial. Lancet.

[42] Bellmunt J, de Wit R, Vaughn DJ, Fradet Y, Lee JL, Fong L, Vogelzang NJ, Climent MA, Petrylak DP, Choueiri TK (2017). Pembrolizumab as second-line therapy for advanced urothelial carcinoma. N Engl J Med.

[43] Killock D (2017). Lung cancer: frontline nivolumab - CheckMate 026 ends in stalemate. Nat Rev Clin Oncol.

[44] Brahmer J, Reckamp KL, Baas P, Crino L, Eberhardt WE, Poddubskaya E, Antonia S, Pluzanski A, Vokes EE, Holgado E (2015). Nivolumab versus docetaxel in advanced squamous-cell non-small-cell lung cancer. N Engl J Med.

[45] Carbone DP, Reck M, Paz-Ares L, Creelan B, Horn L, Steins M, Felip E, van den Heuvel MM, Ciuleanu TE, Badin F (2017). First-Line nivolumab in stage IV or recurrent non-small-cell lung cancer. N Engl J Med.

[46] Friedman J, Moore EC, Zolkind P, Robbins Y, Clavijo PE, Sun L, Greene S, Morisada MV, Mydlarz WK, Schmitt N (2020). Neoadjuvant PD-1 immune checkpoint blockade reverses functional immunodominance among tumor antigen-specific T cells. Clin Cancer Res.

[47] Hodi FS, Chesney J, Pavlick AC, Robert C, Grossmann KF, McDermott DF, Linette GP, Meyer N, Giguere JK, Agarwala SS (2016). Combined nivolumab and ipilimumab versus ipilimumab alone in patients with advanced melanoma: 2-year overall survival outcomes in a multicentre, randomised, controlled, phase 2 trial. Lancet Oncol.

[48] Hodi FS, Chiarion-Sileni V, Gonzalez R, Grob JJ, Rutkowski P, Cowey CL, Lao CD, Schadendorf D, Wagstaff J, Dummer R (2018). Nivolumab plus ipilimumab or nivolumab alone versus ipilimumab alone in advanced melanoma (CheckMate 067): 4-year outcomes of a multicentre, randomised, phase 3 trial. Lancet Oncol.

[49] de Malet A, Antoni G, Collins M, Soularue E, Marthey L, Vaysse T, Coutzac C, Chaput N, Mateus C, Robert C, Carbonnel F (2019). Evolution and recurrence of gastrointestinal immune-related adverse events induced by immune checkpoint inhibitors. Eur J Cancer.

[50] Su Q, Zhang XC, Wang DY, Zhang HR, Zhu C, Hou YL, Liu JL, Gao ZH (2018). The risk of immune-related endocrine disorders associated with anti-PD-1 inhibitors therapy for solid tumors: a systematic review and meta-analysis. Int Immunopharmacol.

[51] Gu L, Khadaroo PA, Su H, Kong L, Chen L, Wang X, Li X, Zhu H, Zhong X, Pan J, Chen M (2019). The safety and tolerability of combined immune checkpoint inhibitors (anti-PD-1/PD-L1 plus anti-CTLA-4): a systematic review and meta-analysis. BMC Cancer.

[52] Cao JX, Zhang XY, Liu JL, Li JL, Liu YS, Wang M, Xu BL, Wang ZX (2015). Validity of combination active specific immunotherapy for colorectal cancer: a meta-analysis of 2993 patients. Cytotherapy.

[53] Huang Q, Zhang H, Hai J, Socinski MA, Lim E, Chen H, Stebbing J (2018). Impact of PD-L1 expression, driver mutations and clinical characteristics on survival after anti-PD-1/PD-L1 immunotherapy versus chemotherapy in non-small-cell lung cancer: a meta-analysis of randomized trials. Oncoimmunology.

[54] Postow MA, Chesney J, Pavlick AC, Robert C, Grossmann K, McDermott D, Linette GP, Meyer N, Giguere JK, Agarwala SS (2015). Nivolumab and ipilimumab versus ipilimumab in untreated melanoma. N Engl J Med.

[55] Ramos-Esquivel A, van der Laat A, Rojas-Vigott R, Juarez M, Corrales-Rodriguez L (2017). Anti-PD-1/anti-PD-L1 immunotherapy versus docetaxel for previously treated advanced non-small cell lung cancer: a systematic review and meta-analysis of randomised clinical trials. ESMO Open.

[56] Chaput N, Lepage P, Coutzac C, Soularue E, Le Roux K, Monot C, Boselli L, Routier E, Cassard L, Collins M (2017). Baseline gut microbiota predicts clinical response and colitis in metastatic melanoma patients treated with ipilimumab. Ann Oncol.

[57] Frankel AE, Coughlin LA, Kim J, Froehlich TW, Xie Y, Frenkel EP, Koh AY (2017). Metagenomic shotgun sequencing and unbiased metabolomic profiling identify specific human gut microbiota and metabolites associated with immune checkpoint therapy efficacy in melanoma patients. Neoplasia.

[58] Gopalakrishnan V, Spencer CN, Nezi L, Reuben A, Andrews MC, Karpinets TV, Prieto PA, Vicente D, Hoffman K, Wei SC (2018). Gut microbiome modulates response to anti-PD-1 immunotherapy in melanoma patients. Science.

[59] Matson V, Fessler J, Bao R, Chongsuwat T, Zha Y, Alegre ML, Luke JJ, Gajewski TF (2018). The commensal microbiome is associated with anti-PD-1 efficacy in metastatic melanoma patients. Science.

[60] Routy B, Gopalakrishnan V, Daillere R, Zitvogel L, Wargo JA, Kroemer G (2018). The gut microbiota influences anticancer immunosurveillance and general health. Nat Rev Clin Oncol.

[61] Routy B, Le Chatelier E, Derosa L, Duong CPM, Alou MT, Daillere R, Fluckiger A, Messaoudene M, Rauber C, Roberti MP (2018). Gut microbiome influences efficacy of PD-1-based immunotherapy against epithelial tumors. Science.

[62] Temraz S, Nassar F, Nasr R, Charafeddine M, Mukherji D, Shamseddine A (2019). Gut microbiome: a promising biomarker for immunotherapy in colorectal cancer. Int J Mol Sci.

[63] Sivan A, Corrales L, Hubert N, Williams JB, Aquino-Michaels K, Earley ZM, Benyamin FW, Lei YM, Jabri B, Alegre ML (2015). Commensal Bifidobacterium promotes antitumor immunity and facilitates anti-PD-L1 efficacy. Science.

[64] Ivanov II, Atarashi K, Manel N, Brodie EL, Shima T, Karaoz U, Wei D, Goldfarb KC, Santee CA, Lynch SV (2009). Induction of intestinal Th17 cells by segmented filamentous bacteria. Cell.

[65] Grangette C (2012). Bifidobacteria and subsets of dendritic cells: friendly players in immune regulation!. Gut.

[66] Marcobal A, Barboza M, Sonnenburg ED, Pudlo N, Martens EC, Desai P, Lebrilla CB, Weimer BC, Mills DA, German JB, Sonnenburg JL (2011). Bacteroides in the infant gut consume milk oligosaccharides via mucus-utilization pathways. Cell Host Microbe.

[67] Vetizou M, Daillere R, Zitvogel L (2016). The role of intestinal microbiota in the response to anti-tumor therapies. Med Sci (Paris).

[68] Frankel AE, Deshmukh S, Reddy A, Lightcap J, Hayes M, McClellan S, Singh S, Rabideau B, Glover TG, Roberts B, Koh AY (2019). Cancer immune checkpoint inhibitor therapy and the gut microbiota. Integr Cancer Ther.

[69] Wang G, Yu Y, Wang YZ, Wang JJ, Guan R, Sun Y, Shi F, Gao J, Fu XL (2019). Role of SCFAs in gut microbiome and glycolysis for colorectal cancer therapy. J Cell Physiol.

[70] Gopalakrishnan V, Helmink BA, Spencer CN, Reuben A, Wargo JA (2018). The influence of the gut microbiome on cancer, immunity, and cancer immunotherapy. Cancer Cell.

[71] Milano G (2019). Efficacy of immunotherapy, gut microbiota and impact of antibiotic use: are there confounding factors?. Cancer Chemother Pharmacol.

[72] Pinato DJ, Howlett S, Ottaviani D, Urus H, Patel A, Mineo T, Brock C, Power D, Hatcher O, Falconer A (2019). Association of prior antibiotic treatment with survival and response to immune checkpoint inhibitor therapy in patients with cancer. JAMA Oncol.

[73] Wang H, Zhou J, Guo X, Li Y, Duan L, Si X, Zhang L (2020). Use of glucocorticoids in the management of immunotherapy-related adverse effects. Thorac Cancer.

[74] Reed JP, Devkota S, Figlin RA (2019). Gut microbiome, antibiotic use, and immunotherapy responsiveness in cancer. Ann Transl Med.

[75] van de Garde EMW, van Bedaf LR, Hurkmans DP, van den Heuvel MM (2020). Antibiotic use and reduced effectiveness of second-line immunotherapy for lung cancer: all the time or just at the start of treatment?. Ann Oncol.

[76] Kulkarni AA, Ebadi M, Zhang S, Meybodi MA, Ali AM, DeFor T, Shanley R, Weisdorf D, Ryan C, Vasu S (2020). Comparative analysis of antibiotic exposure association with clinical outcomes of chemotherapy versus immunotherapy across three tumour types. ESMO Open.

[77] Tinsley N, Zhou C, Tan G, Rack S, Lorigan P, Blackhall F, Krebs M, Carter L, Thistlethwaite F, Graham D, Cook N (2020). Cumulative antibiotic use significantly decreases efficacy of checkpoint inhibitors in patients with advanced cancer. Oncologist.

[78] Pinato DJ, Gramenitskaya D, Altmann DM, Boyton RJ, Mullish BH, Marchesi JR, Bower M (2019). Antibiotic therapy and outcome from immune-checkpoint inhibitors. J Immunother Cancer.

[79] Hakozaki T, Okuma Y, Omori M, Hosomi Y (2019). Impact of prior antibiotic use on the efficacy of nivolumab for non-small cell lung cancer. Oncol Lett.

[80] Sen S, Carmagnani Pestana R, Hess K, Viola GM, Subbiah V (2018). Impact of antibiotic use on survival in patients with advanced cancers treated on immune checkpoint inhibitor phase I clinical trials. Ann Oncol.

[81] Huemer F, Rinnerthaler G, Westphal T, Hackl H, Hutarew G, Gampenrieder SP, Weiss L, Greil R (2018). Impact of antibiotic treatment on immune-checkpoint blockade efficacy in advanced non-squamous non-small cell lung cancer. Oncotarget.

[82] Ahmed J, Kumar A, Parikh K, Anwar A, Knoll BM, Puccio C, Chun H, Fanucchi M, Lim SH (2018). Use of broad-spectrum antibiotics impacts outcome in patients treated with immune checkpoint inhibitors. Oncoimmunology.

[83] Derosa L, Routy B, Kroemer G, Zitvogel L (2018). The intestinal microbiota determines the clinical efficacy of immune checkpoint blockers targeting PD-1/PD-L1. Oncoimmunology.

[84] Becattini S, Taur Y, Pamer EG (2016). Antibiotic-induced changes in the intestinal microbiota and disease. Trends Mol Med.

[85] Huang L, Chen X, Zhou L, Xu Q, Xie J, Zhan P, Lv T, Song Y (2021). Antibiotic exposure windows and the efficacy of immune checkpoint blockers in patients with cancer: a meta-analysis. Ann Palliat Med.

[86] Hiraoka N, Onozato K, Kosuge T, Hirohashi S (2006). Prevalence of FOXP3+ regulatory T cells increases during the progression of pancreatic ductal adenocarcinoma and its premalignant lesions. Clin Cancer Res.

[87] Pushalkar S, Hundeyin M, Daley D, Zambirinis CP, Kurz E, Mishra A, Mohan N, Aykut B, Usyk M, Torres LE (2018). The pancreatic cancer microbiome promotes oncogenesis by induction of innate and adaptive immune suppression. Cancer Discov.

[88] Zhu J, de Tenbossche CGP, Cane S, Colau D, van Baren N, Lurquin C, Schmitt-Verhulst AM, Liljestrom P, Uyttenhove C, Van den Eynde BJ (2017). Resistance to cancer immunotherapy mediated by apoptosis of tumor-infiltrating lymphocytes. Nat Commun.

[89] Zitvogel L, Ma Y, Raoult D, Kroemer G, Gajewski TF (2018). The microbiome in cancer immunotherapy: diagnostic tools and therapeutic strategies. Science.

[90] Sethi V, Vitiello GA, Saxena D, Miller G, Dudeja V (2019). The role of the microbiome in immunologic development and its implication for pancreatic cancer immunotherapy. Gastroenterology.

[91] Kaderbhai C, Richard C, Fumet JD, Aarnink A, Foucher P, Coudert B, Favier L, Lagrange A, Limagne E, Boidot R, Ghiringhelli F (2017). Antibiotic use does not appear to influence response to nivolumab. Anticancer Res.

[92] Elkrief A, El Raichani L, Richard C, Messaoudene M, Belkaid W, Malo J, Belanger K, Miller W, Jamal R, Letarte N (2019). Antibiotics are associated with decreased progression-free survival of advanced melanoma patients treated with immune checkpoint inhibitors. Oncoimmunology.

[93] Giuliani J, Bonetti A (2019). Immunotherapy in first-line for advanced non-small cell lung cancer: <br>a cost-effective choice?. Recenti Prog Med.

[94] Iglesias-Santamaria A (2020). Impact of antibiotic use and other concomitant medications on the efficacy of immune checkpoint inhibitors in patients with advanced cancer. Clin Transl Oncol.

[95] Mohiuddin JJ, Chu B, Facciabene A, Poirier K, Wang X, Doucette A, Zheng C, Xu W, Anstadt EJ, Amaravadi RK (2020). Association of antibiotic exposure with survival and toxicity in patients with melanoma receiving immunotherapy. J Natl Cancer Inst.

